# Analyzing and Modeling the Dysfunction of Inhibitory Neurons in Alzheimer’s Disease

**DOI:** 10.1371/journal.pone.0168800

**Published:** 2016-12-30

**Authors:** Carlos Perez, Jokubas Ziburkus, Ghanim Ullah

**Affiliations:** 1 Department of Physics, University of South Florida, Tampa, FL 33620, United States of America; 2 Department of Biology and Biochemistry, University of Houston, Houston, TX 77204, United States of America; Georgia State University, UNITED STATES

## Abstract

Alzheimer’s disease (AD) is characterized by the abnormal proteolytic processing of amyloid precursor protein, resulting in increased production of a self-aggregating form of beta amyloid (A*β*). Several lines of work on AD patients and transgenic mice with high A*β* levels exhibit altered rhythmicity, aberrant neuronal network activity and hyperexcitability reflected in clusters of hyperactive neurons, and spontaneous epileptic activity. Recent studies highlight that abnormal accumulation of A*β* changes intrinsic properties of inhibitory neurons, which is one of the main reasons underlying the impaired network activity. However, specific cellular mechanisms leading to interneuronal dysfunction are not completely understood. Using extended Hodgkin-Huxley (HH) formalism in conjunction with patch-clamp experiments, we investigate the mechanisms leading to the impaired activity of interneurons. Our detailed analysis indicates that increased Na^+^ leak explains several observations in inhibitory neurons, including their failure to reliably produce action potentials, smaller action potential amplitude, increased resting membrane potential, and higher membrane depolarization in response to a range of stimuli in a model of APP_*SWE*_/PSEN1DeltaE9 (APdE9) AD mice as compared to age-matched control mice. While increasing the conductance of hyperpolarization activated cyclic nucleotide-gated (HCN) ion channel could account for most of the observations, the extent of increase required to reproduce these observations render such changes unrealistic. Furthermore, increasing the conductance of HCN does not account for the observed changes in depolarizability of interneurons from APdE9 mice as compared to those from NTG mice. None of the other pathways tested could lead to all observations about interneuronal dysfunction. Thus we conclude that upregulated sodium leak is the most likely source of impaired interneuronal function.

## Introduction

Alzheimer’s disease (AD) is a fatal neurodegenerative disease that leads to cognitive, memory, and behavioral impairments followed by progressive cell death. The symptoms of AD include the extracellular deposition of beta amyloid (A*β*) plaques and intracellular neurofibrillary tangles—aggregates of microtubule-associated protein *τ* [1]. According to the amyloid hypothesis, the accumulation of A*β* oligomers or plaques due to the imbalance between synthesis and clearance as a result of abnormal processing of amyloid precursor protein (APP) is the driving force for AD pathogenesis [2]. While the exact mechanisms are not entirely known, extensive research suggests the accumulation of A*β* as a critical contributor to the development of early cognitive dysfunctions, such as memory loss, seen in the early stages of AD [3].

Pathological levels of A*β* have been linked to the disruption of synaptic function and the mechanisms responsible for learning and memory. For example, the acute application of A*β* oligomers has been correlated with a decline in long term potentiation [4–7], enhanced synaptic depression [8–10], and cognitive impairments [11, 12]. Details about the effects of excessive A*β* levels on the neuronal networks and as a result the impairment of their function are slowly emerging. Neurons located near A*β* plaques are shown to have enhanced neural activity that may result from a decrease in synaptic inhibition [13]. Transgenic animal lines exhibit spontaneous epileptiform activity [14, 15] and the incidences of epileptic seizures are also increased in AD patients [15, 16]. Similarly, the sleep/wake cycle is markedly disrupted with an increase in wakefulness associated with a decrease in the slow oscillation responsible for non-rapid eye movement sleep rhythms [17, 18]. Gamma [12] as well as beta rhythms [19] are also altered in AD. Despite strong evidence in favor of impaired neuronal network activity, the mechanism leading to such network behavior is incompletely understood [20].

Several studies have attributed the altered neuronal network activity to the dysfunction of inhibitory neurons. The application of *γ*-aminobutyric acid type A (GABA_*A*_) agonist diazepam markedly reduced the activity of hyperactive neurons near A*β* plaques suggesting that an impaired synaptic inhibition rather than intrinsic firing of excitatory neurons underlies the hyperactivity [13]. Due to their key role in gamma rhythm, Verret et al [12] investigated parvalbumin inhibitory neurons (PV) in detail and found that the impairment of these cells leads to the observed spontaneous epileptiform activity, hypersynchrony, and reduced gamma oscillatory activity in human APP (hAPP) transgenic mice and AD patients. In line with these observations we recently reported the failure of inhibitory neurons to reliably fire action potentials leading to hippocampal dysfunction and profound disruptions in dentate gyrus (DG) circuit activity in APP_*SWE*_/PSEN1DeltaE9 (APdE9) aged mouse model of AD [10]. All these observations highlight the importance of the aberrant inhibitory neurons’ activity in the early stages of AD and beg the key question: how do the pathological levels of A*β* oligomers mediate the impairment of inhibitory neurons?

In this study, we use an augmented Hodgkin-Huxley formalism incorporating dynamic ion concentrations inside and outside the inhibitory neuron in conjunction with patch-clamp experiments to identify the pathways leading to impaired inhibitory neuronal activity in the hippocampus of aged mice model of AD. Our previous observations show that inhibitory neurons from APdE9 mice cannot reliably fire action potentials and have higher resting membrane potentials as compared to those from non-transgenic (NTG) mice. Therefore, we use the number of spikes in response to 500 ms long stimulus and the value of the resting membrane potential as initial criteria for investigating the mechanism responsible for aberrant interneuronal activity. Elevating the conductance of sodium leak channels (${\text{G}}_{Na}^{L}$) two to five fold and hyperpolarization activated h-channel ten to hundred fold as compared to interneurons from NTG mice results in the observed number of spikes and resting membrane potential in interneurons from transgenic mice. No other pathways included in our model lead to the observations in both the number of spikes and resting membrane potential. However, there is strong experimental evidence in favor of a reduced density of voltage gated sodium channels (VGSCs) in tissues from hAPP transgenic mice and AD patients [12]. We therefore included a detailed analysis of the effect of changes in the VGSCs conductance (${\text{G}}_{Na}^{F}$) on the behavior of interneurons. While changing ${\text{G}}_{Na}^{F}$ results in the observed number of spikes and other behaviors, it fails to reproduce the higher resting membrane potential in interneurons from APdE9 mice model. Our detailed analysis taking into account several other observations implicates the upregulated sodium leak as the most likely source of impaired interneuronal function.

## Materials and Methods

### Experimental methods

*Animals:* Full details of the experimental procedures and protocols are given in [10]. Briefly, studies were performed on 12-16 month old female mice with mutant human APdE9 and age-matched NTG siblings. These animals are significantly impaired in spatial memory performance by 12 months in the absence of cell death.

*Ethics Statement:* This study was carried out in strict accordance with the recommendations in the Guide for the Care and Use of Laboratory Animals of the National Institutes of Health. The protocol (Permit Number: 08-035) was approved by the University of Houston’s International Animal Care and Use Committee.

*Entorhinal cortical-hippocampal slice preparation:* The mice were anaesthetized with isoflurane and decapitated, and the brains were rapidly excised and placed in oxygenated (95% O_2_-5% CO_2_), ice-cold dissection buffer solution containing (in mM) 212.7 sucrose, 2.5 KCl, 1.25 NaH_2_PO_4_, 3 MgSO_4_, 10 MgCl_2_, 0.5 CaCl_2_, 26 NaHCO_3_, and 10 dextrose. Hippocampal entorhinal cortical slices (350mm) were prepared using a Vibratome (Technical Products International) and preincubated for 0.5 h in normal artificial cerebrospinal fluid (ACSF; pH 7.3, 30uC) containing (in mM): 130 NaCl, 1.2 MgSO_4_, 3.5 KCl, 1.2 CaCl_2_, 10 glucose, 2.5 NaH_2_PO_4_, and 24 NaHCO_3_ aerated with 95%O_2_-5%CO_2_.

*Whole-cell recordings in the aged dentate gyrus interneurons:* To study individual inhibitory neuron activity, we performed whole-cell recordings in the inhibitory cells of the dentate gyrus molecular layer. Inhibitory neurons were visualized and initially identified based on the location and shape of their somatas using infrared optics. For the whole cell current-clamp recordings, micropipettes (4 -7 MΩ) contained: 116 mM K-gluconate, 6 mM KCl, 0.5 mM EGTA, 20 mM HEPES, 10 mM phosphocreatine, 0.3 mM NaGTP, 2 mM NaCl, 4 mM MgATP, and 0.3% neurobiotin (pH 7.25, 295 milli-osmolar). All electrical recordings were performed using MCC 700 amplifiers (Axon Instruments). Whole-cell data were low-pass filtered at 4 kHz and digitized at 10 kHz (Digidata; pCLAMP; Molecular Devices). Passive and active neuronal membrane properties were studied using incremental hyperpolarizing and depolarizing current injections. To elicit spiking activity, depolarizing square wave current pulses incremented by 20 pA were injected into the somas for 500 ms.

### Computational methods

**Fig 1:**
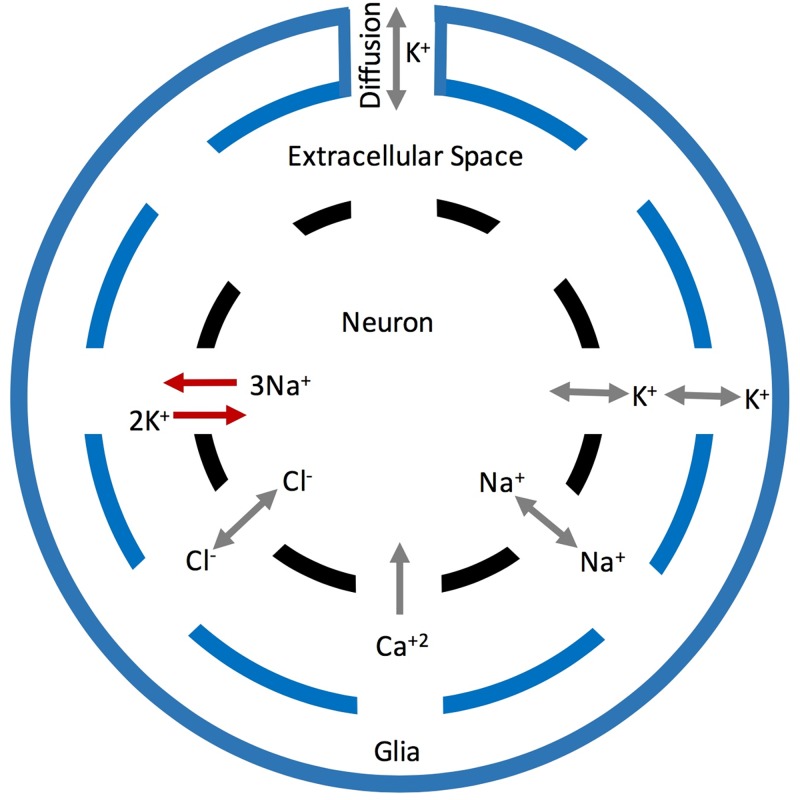
Schematic of the model showing the movement of ions between the neuron, extracellular space, and glia. The gray arrows represent movement of ions between these three spaces due to voltage and ligand-gated channels and the red arrows indicate the current through *Na*^+^/*K*^+^ pumps.

*Membrane potential dyanmics.* The model scheme used in this paper expands on the Hodgen-Huxley formalism and is based on our previous work [21–24] (Fig 1). The change in the membrane potential (V_*m*_) with respect to time is given by contributions from active and passive membrane currents (I_*m*_), applied stimulus (I_*stim*_), and ion transport through Na^+^/K^+^ exchange pumps (I_*pump*_) consuming 1 ATP to extrude three Na^+^ and bring in two K^+^ ions.

$$C\frac{dV_{m}}{dt}=I_{m}+I_{stim}+I_{pump}/\gamma .$$ (1)

*I*_*m*_ is given by contributions from total K^+^ current (*I*_*K*_), total Na^+^ current (*I*_*Na*_), hyperpolarization activated current (*I*_*h*_), Cl^−^ leak current ($I_{Cl}^{L}$), and voltage-gated Ca^2+^ current (*I*_*Ca*_). That is,

$$I_{m}=-\left(I_{K}+I_{Na}+I_{h}+I_{Cl}^{L}+I_{Ca}\right)$$ (2)

where

$$\begin{array}{ll} I_{K} & =I_{K}^{DR}+I_{K}^{M}+I_{K}^{A}+I_{K}^{L}+I_{K}^{Ca}\\ I_{Na} & =I_{Na}^{F}+I_{Na}^{L} \end{array}$$ (3)

The factor *γ* = *S*/(*Fv*_*i*_) is used to convert current unit (*μ*A/cm^2^) into concentration unit (mM/s), where *S*, *v*_*i*_, and *F* are the surface area of the cell, intracellular volume, and Faraday constant. We used a spherical cell with a radius of 6*μ*m. K^+^ currents include delayed rectified ($I_{K}^{DR}$), non-inactivating M ($I_{K}^{M}$), rapidly inactivating A ($I_{K}^{A}$), Ca^2+^ gated ($I_{K}^{Ca}$), and leak ($I_{K}^{L}$) currents. The Na^+^ currents include transient fast ($I_{Na}^{F}$) and leak ($I_{Na}^{L}$) currents. The different K^+^ currents are given as

$$\begin{array}{ll} I_{K}^{DR} & =G_{K}^{F}n^{4}\left(V_{m}-V_{K}\right)\\ I_{K}^{M} & =G_{K}^{M}z\left(V_{m}-V_{K}\right)\\ I_{K}^{A} & =G_{K}^{A}a_{\infty }^{3}b\left(V_{m}-V_{K}\right)\\ I_{K}^{Ca} & =G_{K}^{Ca}c^{2}\left(V_{m}-V_{K}\right)\\ I_{K}^{L} & =G_{K}^{L}\left(V_{m}-V_{K}\right). \end{array}$$ (4)

The two Na^+^ currents are

$$\begin{array}{ll} I_{Na}^{F} & =G_{Na}^{F}m_{\infty }^{3}h\left(V_{m}-V_{Na}\right)\\ I_{Na}^{L} & =G_{Na}^{L}\left(V_{m}-V_{Na}\right). \end{array}$$ (5)

In addition to K^+^ and Na^+^ currents, we have *I*_*h*_, $I_{Cl}^{L}$, and *I*_*Ca*_, which are given as

$$\begin{array}{ll} I_{h} & =G_{h}r\left(V_{m}-V_{h}\right)\\ I_{Cl}^{L} & =G_{Cl}^{L}\left(V_{m}-V_{Cl}\right)\\ I_{Ca} & =G_{Ca}s^{2}\left(V_{m}-V_{Ca}\right). \end{array}$$ (6)

G_*x*_ represents the maximum conductance of a given channel x.

The activation and inactivation variables *a, n, z, b, c, m, r,* and *s* represent the fraction of open or closed channels of different types and are modeled by the rate equations of the form

$$\frac{dq}{dt}=\frac{\left(q_{\infty }-q\right)}{\tau_{q}},q=n,z,b,h,s,r,c.$$ (7)

Where q_∞_ represents the steady state value of the gating variable *q*, and is of the form

$$q_{\infty }=\frac{1}{1+e^{-(V_{m}-\theta )/\sigma }}.$$ (8)

The values for (*θ*, *σ*) in mV are (-30.0, 9.5), (-39.0, 5.0), (-80.0, 6.0), (-50.0, 20.0), (-30.0, 9.5), (-53.0, -7.0), (-84.0, 10.2), and (-20.0, 10.0) for *q*_∞_ = *n*_∞_, *z*_∞_, *b*_∞_, *a*_∞_, *m*_∞_, *h*_∞_, *r*_∞_, and *s*_∞_ respectively. *τ*_*q*_ represents the time constant of a given gate *q*. Since channels responsible for $I_{K}^{Ca}$ are both voltage and ligand gating, the form of its equilibrium value is slightly different and is given as

$$c_{\infty }=\frac{1}{1+\frac{0.03}{48.0{\left({\left[Ca^{2+}\right]}_{i}\right)}^{2}}},$$ (9)

where [Ca^2+^]_*i*_ is the intracellular Ca^2+^ concentration. Time constants for different gating variables are given as,

$$\begin{array}{ll} \tau_{n} & =0.37+1.85\frac{1}{1+e^{\left(V_{m}+27.0\right)/15.0}}\\ \tau_{c} & =\frac{0.2148}{48.0c^{2}+0.03}\\ \tau_{z} & =75.0\\ \tau_{b} & =15.0\\ \tau_{h} & =0.37+2.78\frac{1}{1+e^{\left(V_{m}+40.5\right)/6.0}}\\ \tau_{r} & =\frac{1.0}{e^{-14.59-0.086V_{m}}+e^{-1.87+0.0701V_{m}}}\\ \tau_{s} & =1.0 \end{array}$$ (10)

We assume that the activation of fast Na^+^ and K^+^ A channel is rapid enough so that the instantaneous values of *m* and *a* gates can be used. The reversal potential for Na^+^ (*V*_*Na*_), K^+^ (*V*_*K*_), h (*V*_*h*_), Cl^−^ (*V*_*Cl*_), and Ca^2+^ (*V*_*Ca*_) currents are given by the Nernst equations

$$\begin{array}{ll} V_{Na} & =26.64ln\left(\frac{{\left[Na^{+}\right]}_{o}}{{\left[Na^{+}\right]}_{i}}\right)\\ V_{K} & =26.64ln\left(\frac{{\left[K^{+}\right]}_{o}}{{\left[K^{+}\right]}_{i}}\right)\\ V_{h} & =26.64ln\left(\frac{0.2{\left[Na^{+}\right]}_{o}+{\left[K^{+}\right]}_{o}}{0.2{\left[Na^{+}\right]}_{i}+{\left[K^{+}\right]}_{i}}\right)\\ V_{Cl} & =-26.64ln\left(\frac{{\left[Cl^{-}\right]}_{o}}{{\left[Cl^{-}\right]}_{i}}\right)\\ V_{Ca} & =\frac{26.64}{2}ln\left(\frac{{\left[Ca^{2+}\right]}_{o}}{{\left[Ca^{2+}\right]}_{i}}\right) \end{array}$$ (11)

Where [⋅]_*i*_ and [⋅]_*o*_ represent the concentration of a given ion species in the intra- and extracellular space respectively. The minus sign when computing the Cl^−^ reversal potential is due to its negative charge.

*Ion concentration dynamics.* In addition to membrane potential and different currents, we also keep track of various ion concentrations inside and outside of the interneuron (Fig 1). The change in [K^+^]_*o*_ is a function of *I*_*K*_, *I*_*pump*_, uptake by glia surrounding the neuron (*I*_*glia*_), and diffusion between the neuron and bath perfusate (*I*_*diff*_). The evolution of [Na^+^]_*i*_, is controlled by *I*_*Na*_ and *I*_*pump*_. Finally, the change in [Ca^2+^]_*i*_ is a function of *I*_*Ca*_ and a second term that accounts for the uptake of Ca^2+^ and its gradual return to equilibrium value, [*Ca*^+2^]_∞_ = 50.0 nM.

$$\begin{array}{ll} \frac{d{\left[K^{+}\right]}_{o}}{dt} & =\frac{1}{\tau }\left(\gamma \beta I_{K}-2\beta \gamma I_{pump}-I_{glia}-I_{diff}\right)\\ \frac{d{\left[Na^{+}\right]}_{i}}{dt} & =\frac{1}{\tau }\left(-\gamma I_{Na}-3\gamma I_{pump}\right)\\ \frac{d{\left[Ca^{2+}\right]}_{i}}{dt} & =\frac{1}{\tau }\left(-\gamma I_{Ca}+\frac{{\left[Ca^{2+}\right]}_{\infty }-{\left[Ca^{2+}\right]}_{i}}{\tau_{Ca}}\right) \end{array}$$ (12)

*β* in the above equations is the ratio of intracellular to extracellular volume, *β* = *v*_*i*_/*v*_*o*_, and *τ* = 1000 is used to convert seconds to milliseconds. While the change in [Ca^2+^]_*i*_ is described by the equation above, [Ca^2+^]_*o*_ is fixed at 1.2 mM. [K^+^]_*i*_ and [Na^+^]_*o*_ are linked to [Na^+^]_*i*_ as previously described [21, 23, 25, 26].

$$\begin{array}{ll} {\left[K^{+}\right]}_{i} & =140.0+\left(18.0-{\left[Na^{+}\right]}_{i}\right)\\ {\left[Na^{+}\right]}_{o} & =144.0+\beta \left({\left[Na^{+}\right]}_{i}-18.0\right) \end{array}$$ (13)

The change in intracellular and extracellular volume is negligible and is omitted from the model. [Cl^−^]_*i*_ and [Cl^−^]_*o*_ are given by the conservation of charge inside and outside the cell respectively [23, 25, 26].

$$\begin{array}{ll} {\left[Cl^{-}\right]}_{i} & ={\left[Na^{+}\right]}_{i}+{\left[K^{+}\right]}_{i}+2.0{\left[Ca^{2+}\right]}_{i}-150.0\\ {\left[Cl^{-}\right]}_{o} & ={\left[Na^{+}\right]}_{o}+{\left[K^{+}\right]}_{o}+2.0{\left[Ca^{2+}\right]}_{o} \end{array}$$ (14)

The number 150 in the above equation represents the concentration of impermeable cations. The functions describing *I*_*pump*_, *I*_*glia*_, and *I*_*diff*_ are adopted from Cressman et al. [21], and are given as

$$\begin{array}{ll} I_{pump} & =\frac{\rho }{1.0+e^{\left(25.0-{\left[Na^{+}\right]}_{i}/3\right)}}\frac{1}{1.0+e^{\left(5.5-{\left[K^{+}\right]}_{o}\right)}}\\ I_{glia} & =\frac{G_{glia}}{1.0+e^{\left(\left(18.0-{\left[K^{+}\right]}_{o}\right)/2.5\right)}}\\ I_{diff} & =\epsilon_{k}\left({\left[K^{+}\right]}_{o}-{\left[K^{+}\right]}_{bath}\right) \end{array}$$ (15)

where *ρ*, *G*_*glia*_, *ϵ*_*k*_, and [*K*^+^]_*bath*_ represent maximum Na^+^/K^+^ pump strength, maximum glial *K*^+^ uptake, K^+^ diffusion coefficient, and K^+^ concentration in the bath perfusate respectively. All other parameters not explicitly stated in this section are given in Table 1.

**Table 1:** Values and description of different parameters used in the model.

Parameter	Units	Description
*ρ*	28.09 m*mol*/s	maximum Na^+^/K^+^ pump strength
G_*glia*_	66.67 m*mol*/s	maximum glia uptake
C	1.0 *μ*F/cm^2^	Membrane capacitance
*γ*	1.86 m*mol*/(cm⋅*μ*A)	Conversion factor
*β*	7.0	ratio of intra to extracellular volume
$\text{G}{}_{Cl}^{L}$	0.02 mS/cm^2^	Conductance of leak chloride current
$\text{G}{}_{Na}^{F}$	24.0 mS/cm^2^	Maximal conductance of fast sodium
G_*Ca*_	0.08 mS/cm^2^	Maximal conductance of Calcium current
G_*h*_	0.05 mS/cm^2^	Maximal conductance of h-current
${\text{G}}_{K}^{DR}$	3.0 mS/cm^2^	Maximal conductance of potassium current
${\text{G}}_{K}^{L}$	0.02 mS/cm^2^	Conductance of leak potassium current
${\text{G}}_{K}^{A}$	0.25 mS/cm^2^	Maximal Conductance of A-current
${\text{G}}_{K}^{M}$	1.0 mS/cm^2^	Maximal Conductance of M-current
$\text{G}{}_{K}^{Ca}$	0.55 mS/cm^2^	Maximal Conductance of calcium gated potassium current
$\text{G}{}_{Na}^{L}$	0.07 mS/cm^2^	Conductance of leak sodium current

*Numerical Methods.* The rate equations are solved in fortran 90 using the 4th order Runge-Kutta method, with a time step of 0.01 ms. The analysis and statistics of experimental data is performed in matlab. Codes reproducing key results are available upon request from authors.

## Results

### Experimental Observations

**Fig 2:**
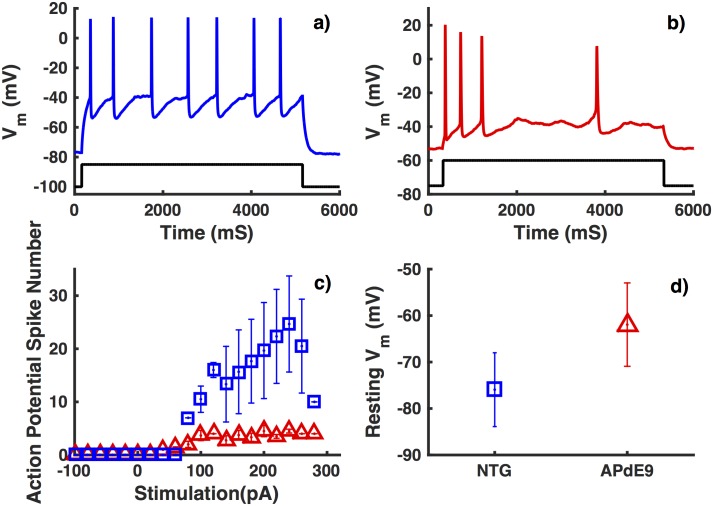
Interneurons from APdE9 mice have impaired spiking ability and higher resting membrane potential as compared to those from NTG mice. Membrane potential in response to an external stimulus of 80 pA (black) observed in interneurons from NTG mice (a) and APdE9 mice (b). Comparison of the number of spikes (c) and mean resting membrane potential (d) in response to 500 ms stimulus of various strengths in interneurons from NTG (squares) and APdE9 mice (triangles). The symbols represent average values from multiple trials. Error bars represent the root mean squared error.

Whole cell recordings in inhibitory neurons from NTG mice display reliable action potential spiking in response to 500 ms stimulus (Fig 2a). Interneurons from APdE9 mice on the other hand are unable to reliably fire action potentials in response to external stimulus (Fig 2b). Interneurons from APdE9 mice show more than 10-fold decrease (depending on stimulus strength) in spiking frequency compared to NTG mice of the same age in response to an external stimulus (Fig 2c). Under resting conditions, interneurons from APdE9 mice are significantly depolarized as compared to NTG mice (resting membrane potential of -77 mV in NTG mice versus -56 mV in APdE9 mice) (Fig 2d).

**Fig 3:**
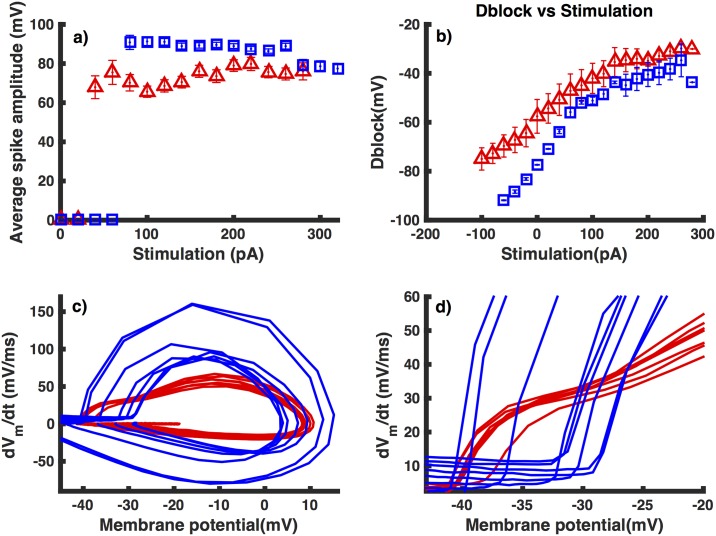
Interneurons from APdE9 mice have smaller mean amplitude, are more depolarized in response to external stimulation, and have different action potential initiation dynamics as compared to interneurons from NTG mice. Mean action potential amplitude (a) and maximum membrane potential during the last 200 ms window of the 500 ms long stimulus after removing the spikes (b) as functions of stimulus strength in interneurons from NTG (squares) and APdE9 (triangles) mice. Action potential in interneurons from NTG mice exhibit rapid onset as compared to those from APdE9 mice. (c) Phase plots showing the derivative of membrane potential as a function of instantaneous membrane potential during action potential in interneurons from NTG (blue) and APdE9 mice (red) observed experimentally. (d) The same phase plots as in (c) but on finer scale to highlight the reduced variability and slow onset of action potentials in interneurons from APdE9 mice as compared to those from NTG mice. Error bars in panels (a) and (b) represent the root mean squared error.

In addition to having smaller frequency and higher resting membrane potential, interneurons from APdE9 mice exhibit action potentials with significantly lower amplitude. At lower stimulation strengths we observe an almost 20 mV decrease in the action potential amplitude (taken in reference to the resting membrane potential) in interneurons from APdE9 mice as compared to those from NTG mice (Fig 3a). The disparity between spiking amplitudes decreases as the applied stimulus increases, eventually converging to almost the same value of 78 mV at I_*stim*_ = 280 pA.

To quantify the depolarizability of the cell we record the maximum value of the membrane potential excluding the spikes during the last 200 ms time window of the 500 ms over which the stimulus is applied. In both APdE9 and NTG mice, the depolarization of the inhibitory neurons on average increases linearly with the stimulus strength for weaker stimulus that is below the threshold for the cell to spike (Fig 3b). The zero stimulus strength in Fig 3b represents the threshold value that is required to result in cell spiking. The depolarization begins to plateau as we increase stimulus strength above the threshold for cell spiking. At negative stimulation strength (-60 pA) interneurons from APdE9 mice exhibit a hyperpolarization of -70 mV as compared to -90 mV in cells from NTG mice. The difference in depolarizability is more pronounced at lower stimulation strengths, and decreases gradually with increasing external stimulus. However, APdE9 mice consistently are more depolarized.

In addition to the differences highlighted above, action potential initiation in interneurons in NTG and APdE9 mice are significantly different. To gain deep insights into the differences in action potential initiation, we quantitatively characterize the dynamics of action potential initiation, which yields important information concerning VGSC activity [27]. We found that action potential initiation in interneurons from NTG mice is characterized by abrupt onset and an upstroke which is much steeper as compared to interneurons from APdE9 mice. This behavior is more clear in the phase plots that graph the rate of change of membrane potential (*dV*_*m*_/*dt*) versus the instantaneous membrane potential and is manifested as almost vertical take-off at the action potential initiation (Fig 3c and 3d). While, the initial kink in the phase plot is similar in the two cases, the rise in *dV*_*m*_/*dt* in case of interneurons from APdE9 mice is biphasic. The biphasic behavior in the initial rise of *dV*_*m*_/*dt* could be due to decreased cooperativity in the gating of VGSCs [28] as a result of their decreased expression or disrupted gating behavior. The biphasic behavior could also reflect structural changes in interneurons in APdE9 mice (see also below). Another salient feature that is apparent from the phase plots is that the action potential onset (the membrane potential at which *dV*_*m*_/*dt* crosses 15*mV*/*ms*) [27] in interneurons from NTG mice varies significantly more as compared to interneurons from APdE9 mice. Interneurons from APdE9 mice display a 5 mV range in onset variability, less than half when compared to interneurons from NTG mice (12 mV) in response to the same range of external stimuli. The lack of cooperativity would also explain the reduced variability in action potential onset [27] in interneurons from APdE9 mice. Furthermore, the action potential onset in interneurons from APdE9 mice is shifted to more negative membrane potential values as compared to interneurons from NTG mice (Fig 3c and 3d). A complete understanding of the dramatic changes in the action potential initiation and testing the prediction about the reduced cooperativity in the gating of VGSCs warants future experiments.

## Computational Results

In the following we will vary different parameters in the model as compared to the parameters set giving the observed behavior in interneurons from NTG mice to search for the pathways that would lead to the two observations: the increase in resting membrane potential and the reduced number of spikes in response to a 500 ms stimulus of different strengths in inhibitory neurons from APdE9 mice as compared to those from NTG mice (see Table 2). The parameters leading to these two trends will be further investigated for other experimental observations.

**Table 2:** The effect of changing the peak conductance of different channels on the spiking ability defined as the number of spikes over a 500ms duration and resting membrane potential of the neuron as compared to the control cell.

Conductance	Spiking	RMP
experimental	decrease	increase
Ca_∞_	no change	no change
$\text{G}{}_{Cl}^{L}$	increase	decrease
$\text{G}{}_{K}^{A}$	decrease	decrease
$\text{G}{}_{K}^{M}$	decrease	decrease
G_*Ca*_	no change	decrease
G_*h*_	decrease	increase
${\text{G}}_{K}^{DR}$	no change	increase
${\text{G}}_{K}^{L}$	decrease	decrease
$\text{G}{}_{Na}^{L}$	decrease	increase
$\text{G}{}_{K}^{Ca}$	no change	no change
$\text{G}{}_{Na}^{F}$	decrease	no change

**Fig 4:**
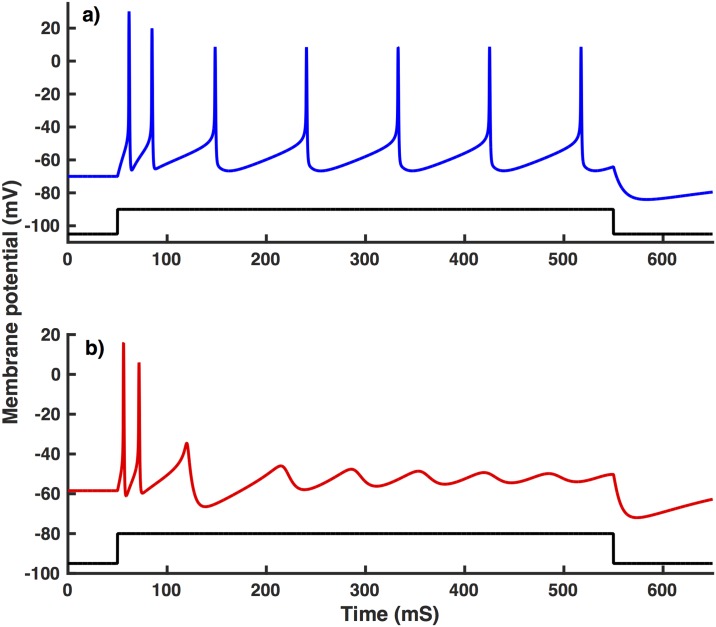
Membrane potential time traces from model inhibitory neurons. Panel (a) shows time trace from the model replicating interneurons from NTG mice using $G_{Na}^{L}=0.007$ mS/cm^2^ and (b) replicates APdE9 mice using $G_{Na}^{L}=0.028$ mS/cm^2^. *I*_*stim*_ = 80 pA was used in these simulations. All other parameters are as given in the text.

**Fig 5:**
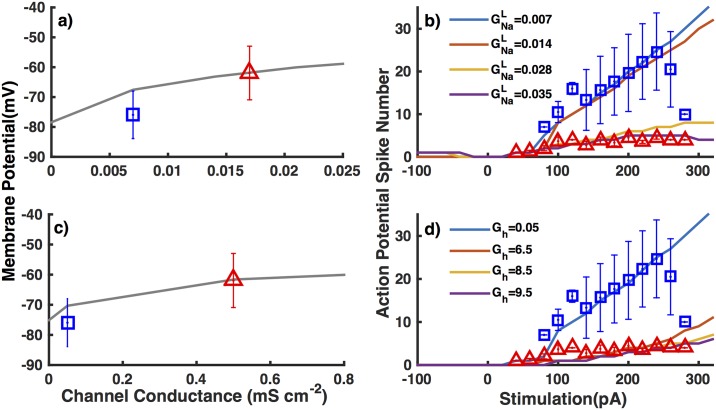
Comparison of resting membrane potential and number of spikes in response to 500 ms stimulus of various strengths in interneurons from NTG and APdE9 mice. Resting membrane potential as a function of $G_{Na}^{L}$ (a) and number of spikes in response to 500 ms of varying stimulus strength for different $G_{Na}^{L}$ values (b) from the model (lines) are compared to the experiment results (symbols). Panels (c) and (d) are the same as (a) and (b) respectively but with varying *G*_*h*_ values. The position of the blue symbol in panels (a) and (c) is adjusted along horizontal axis so that the corresponding $G_{Na}^{L}$ and *G*_*h*_ values reflect these conductances in interneurons from NTG mice.

**Fig 6:**
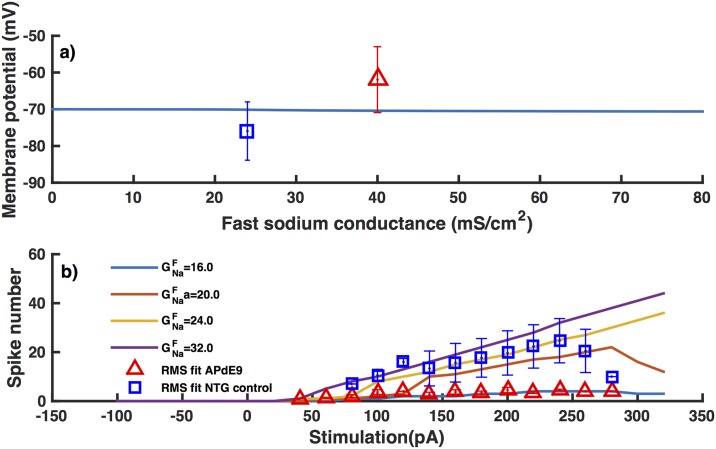
Decreasing $G_{Na}^{F}$ leads to smaller number of spikes but does not change the resting membrane potential. Resting membrane potential as a function of $G_{Na}^{F}$ (a) and number of spikes in response to 500 ms stimulus of varying strengths and different $G_{Na}^{F}$ values (b). Symbols and lines have the same meaning as in Fig (5a) and (5b) respectively except here $G_{Na}^{F}$ is varied instead of $G_{Na}^{L}$.

Increasing $G_{Na}^{L}$ two to five-fold as compared to the value used for interneurons from NTG mice leads to a similar behavior as observed in interneurons from APdE9 mice. Representative time traces for interneurons from NTG and APdE9 mice are shown in Fig 4. A four-fold increase in $G_{Na}^{L}$ is required for the resting membrane potential to be consistent with interneurons from APdE9 mice (Fig 5a). While a five-fold increase leads to the same number of spikes on average in inhibitory neurons from APdE9 mice (Fig 5b). In case of *G*_*h*_ on the other hand, a 10-fold and 130-fold change respectively is necessary to reproduce the observed resting membrane potential (Fig 5c) and number of spikes (Fig 5d) in inhibitory neurons from APdE9 mice. Thus a much higher change in *G*_*h*_ is required to reproduce the observed behaviors. We remark that the number of spikes over the 500 ms duration of stimulus increases proportionally to the stimulus strength for the most part both experimentally and theoretically. The decline in the number of spikes in interneurons from NTG mice at larger stimulation is due to the fact that in one trial the number of spikes is three times smaller than other control data, which has a noticeable effect on the average values. While decreasing $G_{Na}^{F}$ causes a decrease in the number of spikes (Fig 6b), it has negligible effect on the resting membrane potential (Fig 6a).

**Fig 7:**
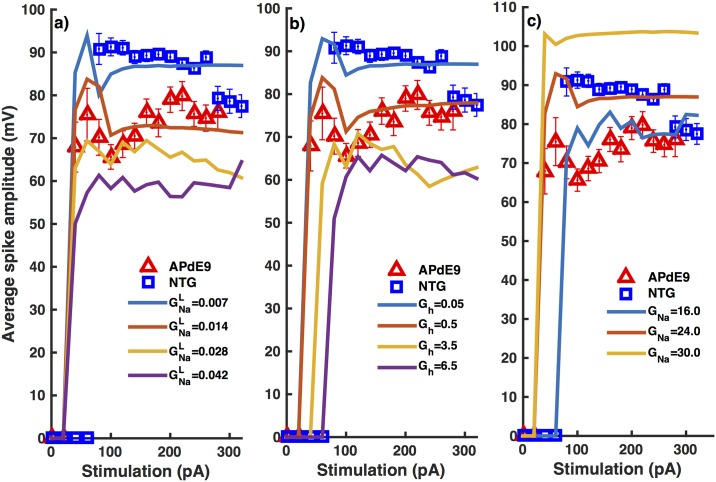
Interneurons from NTG mice exhibit action potentials with significantly higher mean amplitude as a function of stimulus strength as compared to those from APdE9 mice. Change in mean amplitude of all spikes in the time trace as a function of stimulus strength as we vary $G_{Na}^{L}$ (a), *G*_*h*_ (b), and $G_{Na}^{F}$ (c). Symbols and lines represent experimental and theoretical values respectively. Squares and triangles are for interneurons from NTG and APdE9 mice respectively. Error bars represent the root mean squared error.

In addition to decreased number of spikes and higher resting membrane potential, we observe a significant decrease in the average amplitude of all action potentials in inhibitory neurons from APdE9 mice as compared to those from NTG mice (Fig 7). The model agrees closely with the experimental observations and predicts a two-fold increase in $G_{Na}^{L}$ (Fig 7a) and ten-fold increase in *G*_*h*_ (Fig 7b) in interneurons from APdE9 mice as compared to those from NTG mice. Decreasing $G_{Na}^{F}$ from 24.0 mS/cm^2^ (the value giving the same number of spikes in the interneurons from NTG mice) to 16.0 mS/cm^2^ (the value giving the same number of spikes in the interneurons from APdE9 mice) reproduces the observed average amplitude of all action potentials in the series (Fig 7c).

**Fig 8:**
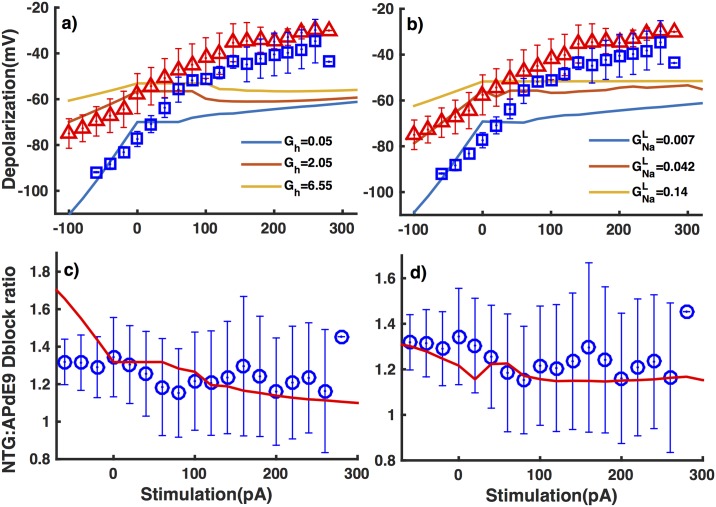
Interneurons from APdE9 mice are more depolarized in response to external stimulation as compared to interneurons from NTG mice. Membrane potential of interneurons during the last 200 ms window of the 500 ms long stimulus after removing the spikes in interneurons from the model as we change *G*_*h*_ (a) and $G_{Na}^{L}$ (b) (lines). Observed values for interneurons from NTG (squares) and APdE9 mice (triangles) are shown for comparison. (c) and (d) are from the same simulations as (a) and (b) respectively except that here we show the ratio of depolarization in interneurons from NTG mice to those from APdE9 mice as a function of stimulus strength (lines and symbols are from the model and experiment respectively).

In line with observations, depolarization linearly increases for a stimulus of -100 to 0 pA in the model and plateaus once the spiking ensues from above *I*_*stim*_ = 0 pA (Fig 8). However, the depolarization in the model plateaus more rapidly as compared to the experiment (Fig 8a and 8b). Our recent modeling study shows that the extent by which a cell can depolarize is significantly affected by the ratio of cell packing in the tissue [23, 26], something not incorporated in the current model. Nevertheless, the model closely reproduces the ratio of the depolarization between inhibitory neurons from NTG and APdE9 mice where a 130-fold and 2-fold increase in *G*_*h*_ and $G_{Na}^{L}$ respectively results in the correct ratio (Fig 8c and 8d). It is important to notice that increasing $G_{Na}^{L}$ by 2-fold as compared to the control value results in the depolarization ratio that agrees well with experimental results for a wide range of stimulus strength. In case of *G*_*h*_ on the other hand, the model exhibits a significantly higher ratio than experiment for lower stimulus strength.

**Fig 9:**
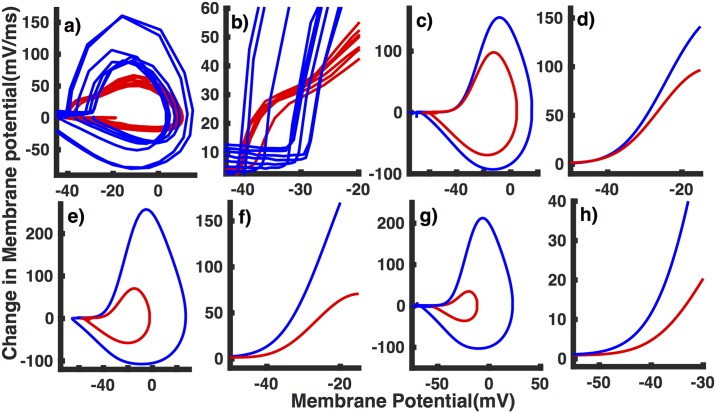
Action potential in interneurons from NTG mice exhibit rapid onset as compared to those from APdE9 mice. (a) Phase plots showing the derivative of membrane potential as a function of instantaneous membrane potential during action potential spike in interneurons from NTG mice (blue) and APdE9 mice (red) observed experimentally are reproduced from Fig 3(c) for comparison. Phase plots given by the model at $G_{Na}^{L}=0.007$ mS/cm^2^ (blue) and $G_{Na}^{L}=0.028$ mS/cm^2^ (red) (c), *G*_*h*_ = 0.05 mS/cm^2^ (blue) and *G*_*h*_ = 6.5 mS/cm^2^ (red) (e), and $G_{Na}^{F}=24$ mS/cm^2^ (blue) and $G_{Na}^{F}=16$ mS/cm^2^ (red) (g) mimicking interneurons from NTG and APdE9 mice respectively. Panels (b), (d), (f), and (h) are extended views of (a), (c), and (e), and (h) respectively.

The model fails to reproduce the observed differences in the action potential onset in cells from NTG and APdE9 mice (Fig 9a and 9b). Increasing $G_{Na}^{L}$ (Fig 9c and 9d) and *G*_*h*_ (Fig 9e and 9f) both cause a shift in the action potential onset towards less negative membrane potential values. We observe a similar rightward shift in action potential onset when $G_{Na}^{F}$ is decreased (Fig 9e and 9f). Although not significant, both $G_{Na}^{L}$ and *G*_*h*_ give the right trend in the variability in the action potential onset (not shown). That is, the range of membrane potential at which the action potential ensues narrows as we increase $G_{Na}^{L}$ and *G*_*h*_. Decreasing *G*_*Na*_ on the other hand leads to a wider range of membrane potential values at the action potential onset, which is not consistent with experimental data (Fig 9a and 9b). Consistent with observations, increasing $G_{Na}^{L}$ and *G*_*h*_ decrease the steepness in the action potential onset. Decreasing $G_{Na}^{F}$ does not change the slope significantly, inconsistent with experimental results.

We remark that in general the action potential onset predicted by the model is significantly slower than observed experimentally, particularly in interneurons from NTG mice. Similarly, the range of onset potential is narrower as compared to observations in NTG mice. The model also fails to reproduce the biphasic nature of the action potential observed in interneurons from APdE9 mice. As pointed out by Naundorf et al [27], models with noncooparative VGSCs are not equipped to replicate the rapid action potential onset and large variability in onset potentials. Replicating both these features simultaneously in interneurons from NTG requires strongly cooperative activation, voltage-dependent inactivation from closed states, and slow recovery from inactivation of VGSCs. As pointed out above, the biphasic nature and reduced variability of action potential onset observed in interneurons from APdE9 mice could also be explained by the reduced cooperativity of VGSCs gating as compared to cells from NTG mice. The multi-compartmental nature of the cell could also lead to biphasic behavior of action potential where the sharp kink results from the axon’s initial segment and the subsequent slower phase is caused by somadendritic compartment [29, 30]. Thus, the switching of action potential onset from being monophasic in NTG mice to biphasic in APdE9 mice could be due to the changes in the morphology or spatial distributions of ion channels in interneurons from brain with AD. Investigating such structural and anatomical changes require spatially explicit models, which is beyond the scope of this study. Nevertheless, the model presented in this paper explains all other observations about the interneurons from both NTG and APdE9 mice. The model also qualitatively exhibits trends that are consistent with the observed changes in the action potential initiation in interneurons form APdE9 mice when $G_{Na}^{L}$ or *G*_*h*_ is increased.

## Discussion

There is strong evidence supporting the theory that the cognitive decline in AD is caused by the dysrhythmic behavior in inhibitory neurons associated with A*β* toxicity [10, 12, 31, 32]. The exact mechanism by which A*β* creates these adverse neuronal defects is not known with any certainty due to the plethora of cellular abnormalities that this protein promotes, such as forming cation-permeable pores in the plasma membrane [33–37], altering channel activity [12, 38, 39], and affecting synaptic signaling [20]. Using Hodgkin-Huxley formalism in conjunction with dynamic ion concentrations, we have reproduced many experimentally observed changes in the behavior of inhibitory neurons from APdE9 mice including the inability to reliably spike, higher resting membrane potential, enhanced depolarizability in response to applied stimulus, and smaller mean action potential amplitude as compared to those from NTG mice. We found that increasing sodium leak and conductance of HCN channels as compared to control values leads to interneuronal characteristics similar to those observed in APdE9 mice. Moreover, while a less than two-fold decrease in $G_{Na}^{F}$ led to the observed number of spikes and mean amplitude of action potentials in interneurons from APdE9 mice, it failed to show the observed trend in the resting membrane potential. None of the other pathways examined lead to the observed interneuronal spiking behavior and resting membrane potential (Table 2). Therefore, we focus our discussion on HCN channels, VGSCs, and *Na*^+^ leak.

There seems to be a strong correlation between elevated levels of A*β* and the activity of HCN channels. A recent experimental study reported significant decrease in the excitability of A*β*-treated pyramidal cells from CA1 region of Hippocampus that was attributed to upregulated *I*_*h*_ current [38]. Saito *et al* on the other hand observed a significant reduction in HCN channel level in the temporal lobe of cynomolgus monkeys during aging and the temporal lobe of sporadic AD patients. The authors speculated that the reduction in the expression of HCN channels may contribute to increased A*β* levels [40]. Although contrasting, these studies point towards a strong correlation between *I*_*h*_ current and neuronal excitability in the presence of excessive A*β* levels. Thus understanding the implications of altered HCN channel activity is an important aspect of elucidating the underlying mechanisms in AD. Increasing the conductance of HCN channels leads to several observations in the inhibitory neurons from APdE9 mice including reduced excitability in line with the observations in [38]. Nevertheless, a more than hundred-fold increase in the conductance of HCN channels is required to reproduce the observed behavior in the interneurons from APdE9 mice—significantly larger than the two to three-fold upregulation at the physiological membrane potential values observed in [38]. We render such dramatic increase unrealistic. Moreover, increasing the conductance of HCN does not account for the observed changes in depolarizability of interneurons from APdE9 mice as compared to those from NTG mice. Thus we conclude that although it might play some role, it is unlikely that the increase in the conductance of HCN channels is the sole cause of all observations in our experiments.

Deficits in VGSCs Nav 1.1 are observed in inhibitory neurons from both AD patients and different animal models of AD. Decreased levels of active Nav 1.1 proteins are believed to be the result of increased *β*-secretase 1 (BACE1) activity, the protein responsible for the cleavage of APP leading to A*β* production as well as the cleavage of the *β*2-subunit of VGSCs, resulting in decreased migration of Nav 1.1 proteins from the intracellular space to the cell membrane [41]. BACE1 levels are elevated in AD patients, and thus it is likely that in addition to promoting increased levels of A*β*, it may also be responsible for the deficit in active Nav 1.1 proteins located in the cell membrane [41, 42]. In experiments performed by Verret *et al* [42] hAPPJ20 mice with decreased levels of the Nav 1.1 protein were observed to exhibit abnormal gamma rhythm, which were closely related to aberrant interneuronal spiking. In addition, *in situ* hybridization measurements support a strong colocalization of Nav 1.1 mRNA to PV neurons, making network hypersynchrony the likely result of abnormal Nav 1.1 expression in inhibitory neurons [12]. Thus there is an abundance of experimental data supporting the hypothesis that abnormal VGSCs cause aberrant neuronal activity in APP and APdE9 mice as well as AD patients [12, 41, 43]. Nevertheless, our results lead us to the conclusion that it is not the only cause of the interneuronal dysfunction. While, nearly halving the maximum conductance of VGSCs resulted in several observations, it failed to capture the increase in the resting membrane potential in the interneurons from APdE9 mice as compared to those from NTG mice. Furthermore, decreasing $G_{Na}^{F}$ led to more variability in the action potential onset values and had no effect on the initial slope of the phase plot. Both these observations are in contradiction to the observed behavior in interneurons from APdE9 mice. Thus, although deficits in VGSCs lead to the smaller mean action potentials and reduced number of spikes, in line with our observations and experiments in [12], they are not the sole cause of the spectrum of aberrant behaviors seen in our experiments.

We suspect that increased *Na*^+^ leak is the major cause of aberrant neuronal behavior in the interneurons from APdE9 mice as it reproduces all observations in our experiments. This enhanced leakage may be due to amyloid pores observed in lipid bilayers exposed to abnormal levels of A*β*. A*β* has been known to form cation-permeable pores in lipid bilayers [33–35], cortical neurons [44], and membranes of other cells [36, 37, 45–47]. Although we are not aware of any evidence showing the formation of A*β* pores in *in vivo* studies, electron microscopy reveals A*β* pore-like structures in cell membranes of post mortem brains of AD patients, but not in control patients [48].

These pores have very high conductance, ranging from 400pS to 4000 pS, allowing large amounts of cations to leak through the membrane [33, 37]. We mimic these pores by increasing the leakage of different cations. While increasing *K*^+^ leak and cytosolic *Ca*^2+^ does not lead to the observed behavior, higher *Na*^+^ leak does reproduce all observations in the interneurons from APdE9 mice. Thus, although most experimental studies focus on the leakage of Ca^2+^ into the cell [34–37], our results suggests that other cations flux through these pores, particularly *Na*^+^ plays a significant role in interneuronal dysfunction.

Physiologically, decreased neuronal spiking due to elevated *Na*^+^ leak could be attributed to a depolarizing shift in the action potential threshold. The threshold for neuronal spiking requires contributions from both non-linear (voltage gated *Na*^+^ and *K*^+^ channels) and linear currents (*Na*^+^ and *K*^+^ leak channels). The addition of these linear currents to the non-linear ones creates an unstable equilibrium point, which results in the spiking threshold we observe during neuronal spiking. Thus a larger contribution from the sodium leak current could shift the spiking threshold value, making it difficult for the the neuron to spike. The decreased amplitude of spiking may be explained by reduced *V*_*Na*_ caused by increased *Na*^+^ leakage, resulting in a smaller action potential during sodium channel activation. In addition to the effects on action potential amplitude and reliability, a more depolarized resting membrane potential could be accounted for by an increased leakage of *Na*^+^ from the extracellular space into the cell, resulting in decreased ionic charge difference, which then causes the resting membrane potential to become more depolarized.

**Fig 10:**
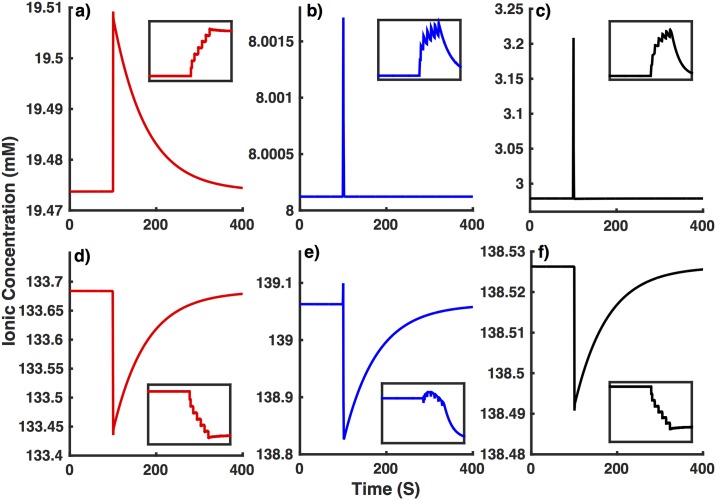
Long-term changes in concentrations of various ionic species in the model interneuron from NTG mice ($G_{Na}^{L}=0.007$ mS/cm^2^) in response to external stimulus during simulations shown in Fig 4a. (a) [Na^+^]_*i*_, (b) [Cl^−^]*_i_*, (c) [K^+^]_*o*_, (d) [Na^+^]_*o*_, (e) [Cl^+^]_0_, and (f) [K^+^]_*i*_. Inset shows enhanced view of changes in ionic concentrations at the time of applied stimulus.

**Fig 11:**
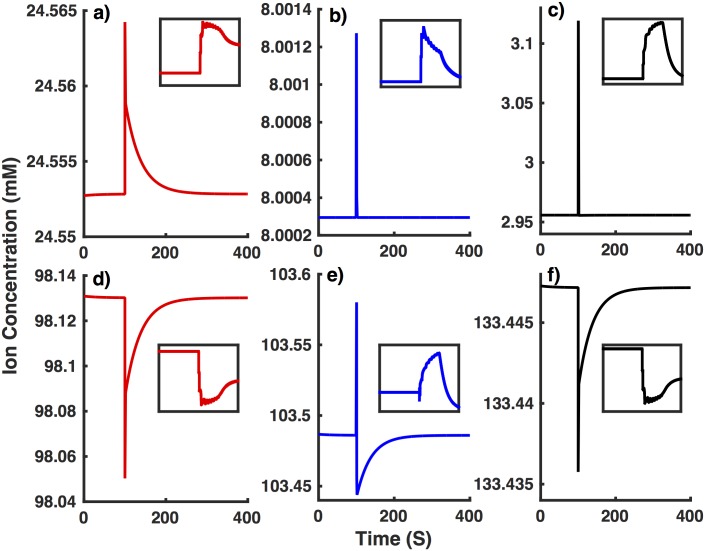
Long-term changes in concentrations of various ionic species in the model interneuron from APdE9 mice ($G_{Na}^{L}=0.028$ mS/cm^2^) in response to external stimulus during simulations shown in Fig 4a. (a) [Na^+^]_*i*_, (b) [Cl^−^]_*i*_, (c) [K^+^]_*o*_, (d) [Na^+^]_*o*_, (e) [Cl^+^]_0_, and (f) [K^+^]_*i*_. Inset shows enhanced view of changes in ionic concentrations at the time of applied stimulus.

This hypothesis is confirmed by the concentrations of various ions in our simulations mimicking interneurons from NTG (Fig 10) and APdE9 (Fig 11) mice. The increased *Na*^+^ leak leads to significantly higher [*Na*^+^]_*i*_ and lower [*Na*^+^]_*o*_ in interneurons from APdE9 mice resulting in lower reversal potential for *Na*^+^ currents (*V*_*Na*_ ∼ 51 mV for NTG versus 37mV for APdE9 mice). This will not only lead to smaller amplitude action potential but also significantly reduce the driving force for *Na*^+^ currents, leaving the cell prone to spiking impairment. Due to the electroneutrality constraint, the lower [*Na*^+^]_*o*_ pulls down [*Cl*^−^]_*o*_. [*Cl*^−^]_*i*_ on the other hand does not change significantly as the increase in [*Na*^+^]_*i*_ is compensated by the decrease in [*K*^+^]_*i*_. The higher [*Cl*^−^]_*o*_ results in more depolarized reversal potential for *Cl*^−^ leak in interneuron from APdE9 mice (*V*_*Cl*_ ∼ -76 mV for NTG versus -68 mV for APdE9 mice). While [*K*^+^]_*i*_ drops by a few mM, the resulting changes in *V*_*K*_ are not strong enough (*V*_*K*_ ∼ -102 mV for NTG versus -101 mV for APdE9 mice) to make major contribution to the spiking behavior and resting membrane potential of the cell. *V*_*h*_ on the other hand decreases from ∼ -42 mV to -48 mV as a result of changes in [*Na*^+^]_*i*_ and [*Na*^+^]_*o*_. This will result in decreased driving force for hyperpolarization-activated current, consistent with the reduced hyperpolarization in response to negative stimulus shown in Fig 8. The decreased hyperpolarization drive together with depolarized *V*_*Cl*_ will lead to higher resting membrane potential in interneurons from APdE9 mice.

While we are unaware of any direct experimental evidence for disrupted *Na*^+^ concentrations, higher resting [*Ca*^2+^]_*i*_ has been observed in neurons from triple transgenic and APP_*SWE*_ mouse models of AD that exhibits accumulation of A*β* oligomers as compared to non-transgenic mice [49]. The fact that the resting [*Ca*^2+^]_*i*_ returned to normal level in the absence of extracellular *Ca*^2+^ and was not restored by blocking voltage gated *Ca*^2+^ channels indicates the possible contribution from influx through A*β* pores. Since A*β* pores are permeable to all cations [45], one can speculate that [*Na*^+^]_*i*_ would also rise. Testing these predictions require future experiments that are beyond the scope of this study.

We also remark that the open probability and permeability of A*β* pores show progressive increase over time [36, 37]. Even the small pores at the early stage have the potential to upregulate the gating of several *Ca*^2+^ channels that could potentially disrupt synaptic function [36]. Big pores at the later stages would severely disrupt cell function due to their significantly higher open probability and conductance. We expect our model to be relevant both at early and later stage of AD. However, it remains to be investigated how the toxicity of pores changes over time *in vivo* and how would they impair interneuronal function in time-dependent manner as they evolve. The increased leak could also trigger synaptic homeostatic process and other intrinsic changes in long term that would require future investigation.

Our model incorporates all key currents that are widely used while modeling inhibitory neurons in the hippocampus (see for example, [50]). Nevertheless, we do not rule out the role of pathways not included in the model in the interneuronal dysfunction. Particularly, our model does not include synaptic conductances that are modulated by A*β*. For example, the application of A*β* to hippocampus slices increases *Ca*^2+^ influx through N-Methyl-D-aspartic acid receptor (NMDAR) [51]. A*β* also blocks *α*7 and *α*4*β*2 subunits of nicotinic acetylcholine receptor (nAChR) in hippocampus and directly evokes sustained nAChR-mediated presynaptic [*Ca*^2+^]_*i*_ increase [52]. Exposing neurons to A*β* enhances the expression of *G*_*q*_ proteins-coupled metabotropic glutamate receptors that generate inositol 1,4,5-trisphosphate (*IP*_3_) [53]. *IP*_3_ and [*Ca*^2+^]_*i*_ act as agonists for *IP*_3_ receptor channel that releases *Ca*^2+^ from the endoplasmic reticulum to the cytoplasm. All these pathways are crucial for understanding the aberrant synaptic signaling and network activity. We believe that our model provides a foundation for building network models to investigate such impairments.

To summarize, our detailed analysis reveals that increased *Na*^+^ leak possibly through the pores formed by A*β* in the plasma membrane leads to nearly all our observations about the interneurons from APdE9 mice. While upregulation of *I*_*h*_ current leads to many observations, we render the required changes in the conductance leading to the observation too high and unrealistic. Similarly, the decreased conductance of VGSCs fails to reproduce the observed depolarized resting membrane potential and cannot be the sole source of interneuronal dysfunction in AD. Our final conclusion is that while restoring the full interneuronal function in AD might require a multifaceted approach, exploring A*β* pore blockers such as NA7 peptide and Bexarotene could lead to promising outcome.
